# Bone mineral density of the proximal femur after hip resurfacing arthroplasty: 1-year follow-up study

**DOI:** 10.1186/1471-2474-12-100

**Published:** 2011-05-19

**Authors:** Arja Häkkinen, Håkan Borg, Mikko Hakulinen, Jukka Jurvelin, Esa Anttila, Tapani Parviainen, Ilkka Kiviranta

**Affiliations:** 1 Department of Physical Medicine and Rehabilitation, Jyväskylä Central Hospital, Jyväskylä, Finland; 2 Department of Health Sciences, University of Jyväskylä, Jyväskylä, Finland; 3 Department of Orthopaedics and Traumatology, University of Helsinki, Helsinki, Finland; 4 Department of Clinical Physiology and Nuclear Medicine, Imaging Center, Kuopio University Hospital, Kuopio, Finland; 5 Department of Physics and Mathematics, University of Eastern Finland, Kuopio, Finland; 6 Department of Orthopaedics and Traumatology, Jyväskylä Central Hospital, Jyväskylä, Finland; 7 Department of Clinical Physiology Jyväskylä Central Hospital, Jyväskylä, Finland

**Keywords:** bone remodeling, dual energy X-ray absorptiometry, stress shielding, stem-neck angle

## Abstract

**Background:**

Hip resurfacing arthroplasty (HRA) is considered a bone-preserving procedure and may eliminate proximal femoral stress shielding and osteolysis. However, in addition to implant-related stress-shielding factors, various patient-related factors may also have an effect on bone mineral density (BMD) of the proximal femur in patients with HRA. Thus, we studied the effects of stem-neck angle, demographic variables, and physical functioning on the BMD of the proximal femur in a one-year follow-up.

**Methods:**

Thirty three patients (9 females and 24 males) with a mean (SD) age of 55 (9) years were included in the study. BMD was measured two days and 3, 6, and 12 months postoperatively and 10 regions of interest (ROI) were used. Stem-neck angle was analyzed from anteroposterior radiographs.

**Results:**

Three months postoperatively, BMD decreased in six out of 10 regions of interest (ROI) on the side operated on and in one ROI on the control side (p < 0.05) compared to the second postoperative day. At 12 months, BMD had increased in 7 ROIs on the operated side and one ROI on the control side (all p < 0.001). Correlation was found between the stem-neck angle and BMD in ROIs 2, 3, 7, and 9 (r = 0.36 - 0.61). In multiple regression analysis, stem-neck angle, age, sex, body mass index, and walking distance did not explain the BMD changes.

**Conclusions:**

After an early drop, the BMD of the upper femur was restored and even exceeded the preoperative level at one year follow-up. From a clinical standpoint, the changes in BMD in these HRA patients could not be explained by stem-neck angle or patient related factors.

## Background

Hip resurfacing arthroplasty (HRA) is a bone conserving procedure compared with the traditional total hip arthroplasty (THA) [1,2]. HRA involves removal of the damaged surfaces of the head of the femur, sparing the femoral neck and acetabulum. In the widely used Birmingham Hip Resurfacing (BHR) design, the femoral component has a short stem. The main part of the femoral head and the femoral neck is saved during the arthroplasty, which it is thought to eliminate the problems of proximal femoral stress shielding and osteolysis and to decrease the dislocation rate [2,3]. The bone saving method also offers the possibility for resurfacing to be converted to a standard THA at a later stage.

However, HRA has also some disadvantages. For example, excessive valgus positioning of the stem or poor operation technique when preparing the femoral head may result in failures [4,5]. The reported failure rates due to femoral neck fracture vary between 0 and 12% [6]. Aseptic loosening of prostheses around the stem and periprosthetic bone loss are thought to be consequences of poor blood supply, stress shielding, and an inflammatory process induced by foreign body particles [4,7].

Although hip resurfacing methods have been used for decades, there are few longitudinal studies about femoral neck bone remodeling. Two studies have shown that bone stock of the femoral neck is preserved when HRA hips are compared to total hip replacement [8] or to hips not operated on [9]. In addition, the valgus positioning of the femoral component was reported to increase compressive stress in the proximal femur and, thus, promote bone remodeling [10].

The purpose of the present prospective study was to quantify the changes in bone remodeling in the upper part of the femur during the first postoperative year after BHR using dual energy X-ray absorptiometry (DEXA). In addition, we sought to study how bone remodeling was affected by stem-neck angle, as well as some patient related factors such as physical activity.

## Methods

In this study 33 consecutive patients (9 females and 24 males) undergoing hip resurfacing surgery were recruited. The mean (SD) age of the participants was 55 (9) years. The mean (SD) body mass index (BMI) before the operation was 28.4 (4.6), and 45% of the participants were overweight (BMI >27) and 21% were obese (BMI >30). The study protocol was explained to the patients and they gave written consent before entering the study. The local ethics committee approved the study protocol. Ethical review committee statement: K-S shp Dnro 50/2003

### DEXA-analysis

The bone mineral density was measured by dual energy X-ray absorptiometry (DEXA) (Lunar Prodigy, GE Healthcare, Madison WI, USA) 2 days and 3, 6, and 12 months postoperatively. All DEXA procedures followed the manufacturer's instructions. During DEXA scans, the patient was in the supine position and patient's limb was positioned in a standard neutral rotation using a supporting device. System quality assurance protocols were performed daily in accordance with the manufacturer's instructions. Both the operated and unoperated hips were scanned simultaneously using the standard dual femur acquisition mode of the instrument.

**Figure 1:**
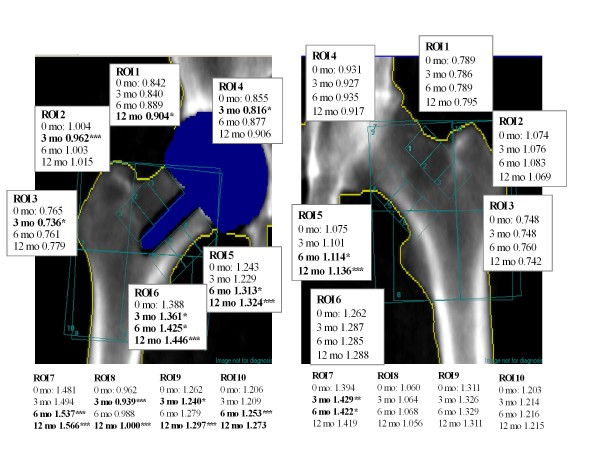
**Mean BMD values (g/cm^2^) in ten regions of interest (ROI) in the operated and control sides during the 12 month follow-up (determined using the dual femur acquisition mode)**. The statistical difference (*p < 0.05, **p < 0.01, and ***p < 0.00) between time-points is indicated.

In the dual femur BMD analysis ten custom made regions of interest (ROI) were defined: ROIs 1-6 were determined according to Kishida et al [8]; ROIs 1-3 corresponded to superolateral zones and ROIs 4-6 to inferior-medial zones. In addition, four larger regions were defined based on Gruen et al [11]: ROI 7 corresponded roughly to Gruen zones 1+7 and ROI 8 to Gruen zones 2+6. ROIs 9 and 10 corresponded to the medial and lateral upper femur including the greater trochanter (Gruen zones 1+2 and 6+7), respectively. Regions of interests were manually adjusted to account for individual anatomy by using a predefined ROI template. DEXA analysis was carried out by an experienced technician (EK) (Figure 1). We have shown the reproducibility of the method to be good [12].

### Radiographs

Preoperative osteoarthritis classification of the hip joints were scored from anterioposterior radiographs (grade 1 = narrowed joint space; grade 2 = narrowed joint space and osteophytes; and grade 3 = narrowed joint space, osteophytes, and severe joint deformation) [13]. The angle between the femoral stem of the prosthesis and the femoral neck (stem-neck angle) was analyzed from the anteroposterior radiographs by an experienced orthopedic surgeon (EA).

Subjectively perceived hip pain during loading, in the week prior to surgery, was assessed by the visual analogue scale (0-100 mm). Self-assessment of walking distance was used and included 3 categories: <500 m; 500 - <1500 m; and >1500 m. The time spent doing leisure physical activities was determined by a questionnaire.

### Operative technique

The patients were operated on by one experienced orthopedic surgeon using a posterior approach with the BHR system [3]. All the patients were allowed to bear full weight on the first post-operative day, although a cane was recommended for 2 weeks after the operation to help with balance.

## Statistical Analysis

The Wilcoxon test for non-parametric comparison of two repeated samples was used for comparing differences between time points (0, 3, 6, and 12 months) and also between the operated and control sides. Correlation coefficients were calculated by the Spearman method. Associations between BMD and the explanatory parameters (stem-neck angle, age, sex, BMI, and walking distance) were analyzed using a logistic regression analysis model.

## Results

The severity of osteoarthritis was grade 2 or 3 in 93% of the patients on the operated side and in 15% on the control side. Hip pain both during loading and at rest decreased significantly on the operated side and was at the same level as the control side at the 12-month follow-up (Table 1). At the same time, the number of participants able to walk 1500 m increased by 72%. The self-reported mean (SD) times spent doing leisure activities preoperatively and at 3 months were 2.3 (2.4) and 2.5 (2.5) hours/week, respectively; but at 12 months, this almost doubled (Table 1).

**Table 1:** Changes in hip pain and physical activity during 12-months follow-up.

	Preoperatively	At 12 months	P-value
Hip pain (VAS, mm), mean (SD)			
During loading			
- operated side	65 (24)	4 (15)	<0.001
- control side	5 (15)	10 (23)	0.23
At rest			
- operated side	36 (27)	1 (5)	<0.001
- control side	7 (17)	6 (17)	0.77
Walking distance ≥1500 m; N (%)	8 (24%)	29 (88%)	<0.001
Leisure time activity h/week, mean (SD)	2.3 (2.4)	4.2 (2.5)	<0.020

In the early postoperative phase, BMD was statistically significantly higher in three ROIs (ROIs 4, 5, and 6) and lower in four ROIs (ROIs 3, 7, 8, and 10) compared to the control side (Figure 1). At the end of the follow-up, BMD was higher in three ROIs (ROIs 5, 6, and 9) and lower in three ROIs (ROIs 2, 7, and 10).

Three months postoperatively, bone mineral density had decreased in six of ten ROIs on the operated side and in one ROI on the control side (p < 0.05) compared to the second postoperative day (Figure 1). At the 12 month follow-up, statistically significant increases in BMD had occurred in 7 ROIs on the operated side and in one ROI on the control side.

In 21 participants, the mean (SD) stem-neck angle was 5.4 (3.4) degrees in valgus and in 12 participants, the angle was 3.6 (3.0) degrees in varus. The range of the angles varied from 11 degrees of varus to 9 degrees of valgus. Positive associations were found between the stem-neck angle shifting from varus to valgus and BMD in ROI 2 (r = 0.51; p = 0.002), ROI 3 (r = 0.36; p = 0.039), ROI 7 (0.61; p < 0.001), and ROI 9 (r = 0.36; p = 0.040). Multiple regression analysis revealed that stem-neck angle, age, sex, BMI, and walking distance were not related to the changes in BMD within ROIs 1-10.

## Discussion

The present study was undertaken to investigate changes in bone remodeling in the upper part of the femur during the first postoperative year after BHR. Our results show that, although three months after operation the BMD levels were inferior in six out of ten ROIs on the operated side compared to preoperative level, the BMD recovered and even exceeded the preoperative level in seven ROIs at the one-year follow-up.

It is widely accepted that BMD of the proximal femur generally decreases after total hip replacement [8,10,14,15]. The reason for this resorption is mainly due to stress-related remodeling during the early postoperative period. On the other hand, the use of the bone conserving HRA method is reported to preserve femoral BMD better than THA due to better load transfer to the proximal femur [16,17]. In addition to implant-related factors, the decrease of BMD can partly be explained by patient related factors like decreased mobility. The present patients were allowed to bear full weight immediately after the operation, although a cane was recommended for 2 weeks afterwards to help with balance. In the study by Lian et al. (2008), partial weight bearing was allowed during the first two weeks, progressing then to full weight bearing over the next two months [10]. As a decrease in bone stock of the proximal femur was found in both of these studies 3 months postoperatively, the small difference in postoperative weight-bearing mobility did not influence bone loss. According to self-reported physical activity diaries, the time spent doing physical activity per day was at the preoperative level at the 3-month follow-up in our patients. This activity level was sufficient to maintain the bone stock of the contra-lateral hip showing that the effect of weight-bearing activities on BMD was smaller than the effect of the surgical procedure.

Another explanation for early bone loss might be impaired vascularization due to HRA [18,6]. Steffen et al. (2005) reported a 62% reduction in femoral head oxygenation during the operation; in some patients, the blood supply was completely lost postoperatively [6]. During resurfacing through a posterior approach, the branches of the medial femoral circumflex artery, which pass along the short external rotators, are likely to be destroyed. This deep branch provides the most important blood supply to the femoral head. In these patients, the posterior approach was used and short rotators were cut. However, the femoral neck was left intact preserving the branches of the femoral circumflex arteries. This should ensure blood and nutrition supplies reach the proximal femur and, thus, prevent bone loss due to impaired vascularization [19]. As the BMD of all ROIs reached at least the preoperative level or even exceeded it, we may assume that blood supply had recovered sufficiently in the present patient sample to support bone remodeling during the one-year follow-up.

In our study, significant new bone stock formation appeared during the one-year follow-up. Thus, hip resurfacing seems to transfer the load to the upper femur, as occurs physiologically. The operation was extremely effective in reducing hip pain during the loading, promoting the patients' walking capacities and physical activity levels. Thus, an increase in weight bearing activities may have, in part, also improved the mechanical loading of the hip. However, the surgical procedure, particularly the division of the external hip rotator muscles, led to specific deficit of external rotation strength and the active range of movements as we have reported earlier [20]. Another recent study also reported HRA patients have significantly decreased hip extension and flexion angles and asymmetric gaits 18 months after surgery [21]. These deficits may increase the risk of dislocations, abnormal lumbopelvic posture, and compensatory motion in the lumbar spine and hips during walking and other daily activities long-term.

Bone remodeling occurred in some extent in all ten ROIs. More specifically, statistically significant increases took place in ROI 1, representing the superolateral zone and in ROIs 5 and 6, representing the inferior-medial zone of the femoral neck. Lian et al. (2008) reported that valgus positioning of the neck is recommended to increase the compressive stress in the femoral head and neck [8]. In our study, there was correlation between stem-neck angle and change in BMD in two superolateral (ROIs 2 and 3) and two larger zones (ROIs 7 and 9), which indicate that the change of stem-angle towards valgus may have some effect on loading of the femoral neck and, thus, on bone remodeling. The stem is important for proper alignment of the femoral component during the operation and the initial stability. However, as the short stem in the femoral neck is situated in cancellous bone, its importance on weight bearing and BMD needs to be researched further.

A limitation of the study is a rather short follow-up time. In other studies published the follow-up times have varied between 6-24 months [6-8]; thus, longer follow-ups with larger patient groups are needed. The strength of the study is that we have repeated the DEXA measurements four times, enabling changes of bone remodeling to be shown within the time frame. We also determined some clinical and functional outcomes of the participants when trying to identify patient-related variables to explain changes in BMD. However, the bone remodeling after HRA may be a result of many simultaneously influencing factors; thus, more research is needed to show these associations.

## Conclusion

Our one-year follow-up showed that there was a drop in bone remodeling in the early postoperative phase after HRA. However, at one-year follow-up, the bone stock of the upper femur was restored and even exceeded the preoperative level. However, analyses showed changes in BMD were not explained by stem-neck angle or patient related factors.

## Competing interests

The study was partly supported by Midland Medical Technologies Nordic.

## Authors' contributions

AH participated in the design of the study, data collection, analysis of the data, and drafting the manuscript. HB participated in design of the study and drafting the manuscript. MH participated in the DEXA analysis and drafting the manuscript. JJ participated in the DEXA analysis and drafting the manuscript. EA participated in data collection, analysis of stem-neck angles, and drafting the manuscript. TP participated in DEXA data collection and drafting the manuscript. IK participated in the design of the study and drafting the manuscript. All authors read and approved the final manuscript.

## Pre-publication history

The pre-publication history for this paper can be accessed here:

http://www.biomedcentral.com/1471-2474/12/100/prepub
